# Predicting Metastasis in Melanoma by Enumerating Circulating Tumor Cells Using Photoacoustic Flow Cytometry

**DOI:** 10.1002/lsm.23286

**Published:** 2020-06-18

**Authors:** Robert H. Edgar, Ahmad Tarhini, Cindy Sander, Martin E. Sanders, Justin L. Cook, John A. Viator

**Affiliations:** 1 Department of Engineering, Duquesne University, Pittsburgh, Pennsylvania 15282; 2 Department of Bioengineering, University of Pittsburgh, 300 Technology Dr, Pittsburgh, Pennsylvania 15213; 3 H. Lee Moffitt Cancer Center and Research Institute, Tampa, Florida 33612; 4 Hillman Cancer Center, University of Pittsburgh Medical Center, 5115 Centre Ave, Pittsburgh, Pennsylvania 15232; 5 Acousys Biodevices Inc., 1777 Highland Drive, Ann Arbor, Michigan 48108

**Keywords:** cancer staging, microfluidic, optoacoustics

## Abstract

**Background and Objectives:**

Enumerating circulating tumor cells has been used as a method of monitoring progression of various cancers. Various methods for detecting circulating melanoma cells (CMCs) have been reported, but none has had sufficient sensitivity to determine if the presence of rare CMCs in the blood of Stage I–III melanoma patients predicts if those patients eventually develop metastatic disease.

**Study Design:**

We quantified CMCs in serial blood samples from 38 early stage melanoma patients to determine if CMC numbers predict development of metastatic melanoma. CMCs were enumerated using a photoacoustic flow cytometric detection system that uses a laser to induce high frequency acoustic signals in pigmented CMCs.

**Results:**

We observed that detection of greater than 2 CMCs/ml of blood from patients with Stage I–III melanoma predicts metastatic disease. Of the 11 patients we studied who had two or fewer CMCs detected at all time points tested, none progressed to metastatic disease over a mean follow-up of 1288 days. In contrast, 18 of the 27 patients (67%) having more than 2 CMCs/ml at one or more time points progressed to metastatic disease over a mean follow-up of 850 days.

**Conclusions:**

Photoacoustic flow cytometry can detect rare CMCs in the blood of Stage I–III melanoma patients and detectionof these cells is predictive of subsequent development of metastatic disease. Lasers Surg. Med.

## INTRODUCTION

The study of circulating tumor cells (CTCs) has been ongoing for many years, with technologies for their detection having varying degrees of success and utility. Detection and capture of CTCs or their cellular components may have utility in monitoring cancer recurrence or progression, and may inform clinicians of the molecular nature of cancer in individual patients with respect to potential drug response [1,2]. More specifically, CTCs have generated much interest with respect to their potential utilization as a diagnostic for detection, capture, and genetic analysis for monitoring for recurrence and progression. A recent study by Lucci et al. reported a strong correlation of CTCs in melanoma patients and relapse of disease. This study was conducted with the CellSearch system, and it was found that more than one CTC in baseline blood samples of recently surgically treated 243 Stage III melanoma patients correlated highly with advancement toward metastasis [3]. Their work paralleled our own findings, though ours were performed on serial sampling of blood from patients. The results of Lucci et al. and our work coincide with many other efforts in CTC research, showing that research interest in CTC assays continues, as a successful method using small blood samples may provide valuable information without departing from current clinical care.

As melanoma metastasis is responsible for the vast majority of skin cancer deaths, the detection and quantification of circulating melanoma cells (CMCs) for monitoring disease relapse may have great utility in patient care [4]. Many efforts for detection and monitoring of melanoma have focused on polymerase chain reaction (PCR) methods, which exploit prior information about DNA and RNA sequences in melanoma. These techniques allow for multiple biomarkers to be assayed, and are supported by substantial industry interest. However, clinical use of PCR detection methods is confounded by their lack of ability to document objectively and quantitatively the actual presence of low numbers of circulating cancer cells in a patient.

Other detection technologies use size filtration or specific antibody-coated surfaces to capture CTCs for later imaging, enumeration, and analysis [5,6,7]. Although there are many antibody-capture techniques for counting CTCs, a label-free method, such as photoacoutic flow cytometry, eliminates the need for predicting the presence of specific cell surface markers in heterogeneous populations of cells. Additionally, some antibody-based detection methods require immobilization of the CTCs on a substrate. Maintaining CTCs in an unbound, suspended state has potential advantages in analyzing cells for various biological functions that might be perturbed by antibodies binding to cell surface molecules capable of transducing signals to the captured cells.

Photoacoustic detection is based on transduction of absorbed light from a laser source into ultrasonic waves. It has the ability to specifically detect materials based on their optical absorption. Materials that produce a robust acoustic signal, such as melanin crystals in melanoma cells, can be detected and analyzed using their photoacoustic properties [8]. Photoacoustic flow cytometry uses this principle to detect rare particles in body fluids in a fluidic system [9,10,11]. This type of flow cytometry is distinct from standard fluorescent flow cytometric methods as the laser excitation can simultaneously irradiate a large number of cells while actively identifying only rare particles, such as melanoma cells in blood, that have specific optical absorption. This advantage makes photoacoustic flow cytometric detection of CMCs rapid and sensitive, allowing detection of a single melanoma cell spiked into a 10 ml sample of blood in only a few minutes of processing time, as previously reported [12].

We have enhanced our photoacoustic flow cytometer to induce two-phase flow in order to enable cell capture for later analysis [12]. This two-phase flow introduces an immiscible fluid, such as mineral oil, to the flow system to create alternating bubbles of cell suspension and oil. This train of droplets is sequentially irradiated with laser light. Bubbles that generate photoacoustic waves are assumed to have cells of interest and are sorted down-stream. Other bubbles are discarded as waste.

We used this sensitive acoustic flow cytometric detection system to assay for CMCs in serial blood samples from 38 Stage I–III melanoma patients. Our goal was to determine if CMC enumeration over time has predictive value for progression to metastatic disease. Our analysis showed a predictive association of detected CMCs with whether patients remained free of metastatic disease, or experienced disease relapse with metastases.

## MATERIALS AND METHODS

### Photoacoustic Flow Cytometry

**Fig. 1.**
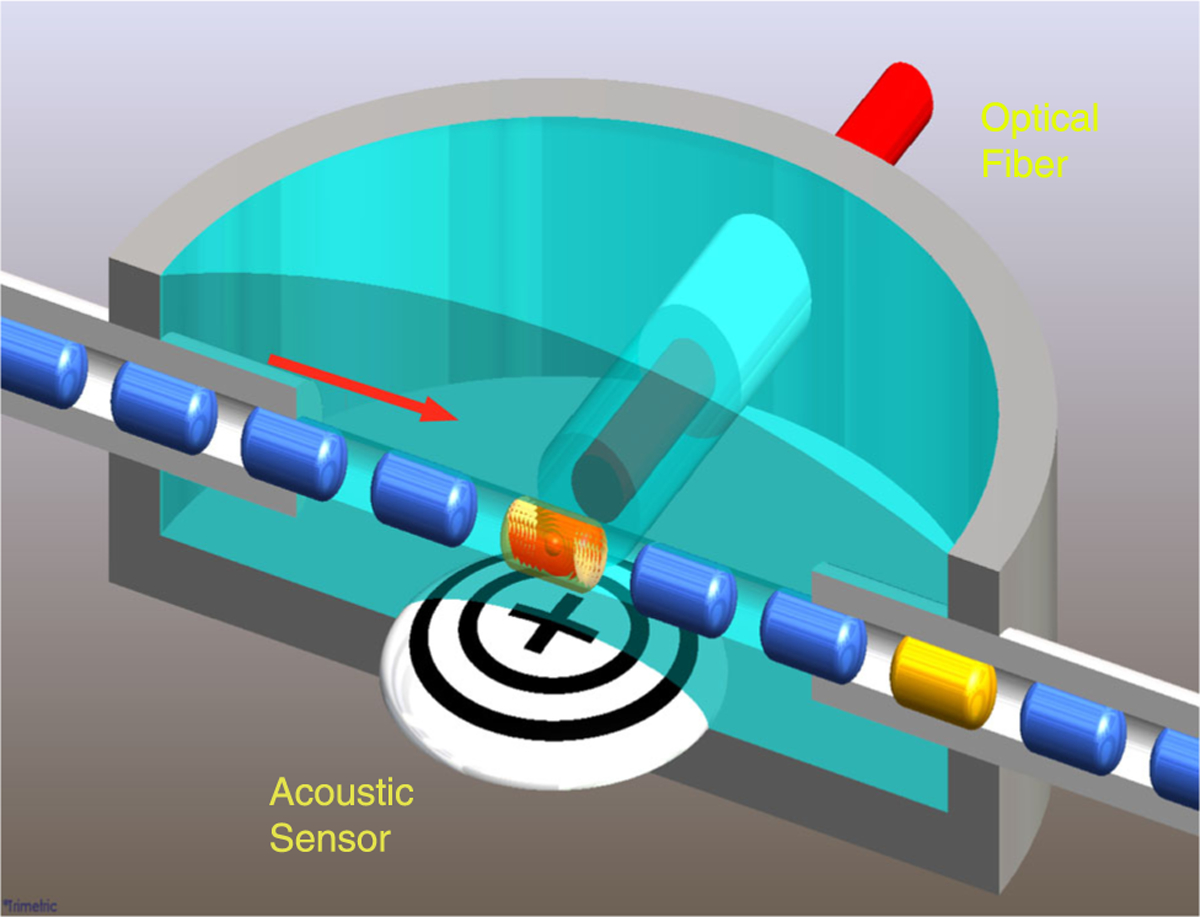
The photoacoustic flow cytometer separates continuous flow of blood cells with air bubbles. The resulting blood cell suspension droplets are irradiated by laser light. Direction of flow of the droplets is indicated by the red arrow. Droplets that contain circulating tumor cells (CTCs) generate photoacoustic waves that are sensed by an acoustic transducer. These droplets are shunted off to a collection cuvette for further analysis. Bubbles that do not generate photoacoustic waves are assumed not to contain CTCs and are diverted for disposal.

Photoacoustic generation is the process of inducing acoustic waves in a medium after irradiation with light. Virtually all photoacoustic methods use laser energy and most modern manifestations used rapid pulse laser systems. While there are several distinct physical processes to induce photoacoustic waves, our method uses thermoelastic expansion [12,13]. This process comes about when rapid laser heating of an optical absorber results in thermal expansion of the material, resulting in pressure waves. It is this process that occurs in photoacoustic flow cytometry, where pulsed laser light gets selectively absorbed by melanoma granules, generating sound waves. In order to detect and enumerate any circulating melanoma cells in the patient samples, we used a Q-switched Nd:YAG laser operating at 532 nm with a pulse duration of 5 nanoseconds to induce acoustic waves in melanoma cells under flow. The system is shown diagrammatically in Figure 1.

Laser light was launched into a 1000 μm, multimode optical fiber with a numerical aperture of 0.39 (Thorlabs, Newton, NJ). The laser energy typically varied from 1.9 to 2.1 mJ. Laser light was directed to a quartz tube through which the sample flowed. This tube had a 10 μm wall thickness. The thin wall enabled maximum transfer of energy from the flow within the tube to an acoustic sensor. The quartz tube was immersed in acoustic-matching gel (Sonotech LithoClear; NEXT Medical Products Company, North Branch, NJ). The optical fiber was bent as a means to mix modes, resulting in a near-Gaussian beam. Fluence was calculated to be 0.014 mJ/cm^2^.

Two syringe pumps were used to create two-phase flow, that is, fluid sample separated by an immiscible mineral oil, as shown in Figure 1. This change from continuous flow allowed for sequestration of cellular material into a small volume. If a melanoma cell was detected by a photoacoustic response, this volume was kept using fluidic capture for later analysis. The syringe pumps created alternating flow of sample and mineral oil equal to a 60 μl/min flow rate. The flow rate and fluid properties resulted in approximately 1000 sample bubbles from the milliliter of sample.

The acoustic waves were sensed by a focused ultrasound transducer with a focal length of 12.5 mm and a center frequency of 5 MHz. These signals were amplified with a gain of 50 using a Tegam 4040B system and was sent to a desktop computer with custom LabVIEW code (National Instruments, Austin, TX) for instrumentation control and data acquisition. Classification of photoacoustic events was performed using a simple threshold. The noise floor was determined by irradiating phosphate-buffered saline (PBS), as there is no optical absorption at 532 nm. The threshold was set at three times the noise floor to classify a signal as originating from a CMC. Comprehensive design and descriptions of the photoacoustic flow cytometer for detection and capture of CMCs are given in O’Brien et al. [12]. The laser beam and the detection volume of the acoustic sensor were aligned to the detection chamber of the photoacoustic flow cytometer. The cell suspension from each sample had approximately 1000 bubbles.

We assumed the number of CMCs in each milliliter sample would be much smaller than 1000, thus, with a well-mixed suspension of cells, two-phase flow would result in most bubbles having no cells. Poisson statistics predicts that there would be a number of bubbles with one CMC, approximately equal to the total number of CMCs. That is, if there are 33 CMCs in a sample, there would be about 32–33 bubbles with one CMC each. There may be a bubble with two or more CMCs, though this possibility would be rare. Poisson statistics is described by

(1) $$P\left(k\right)=e^{-\lambda }\frac{\lambda^{k}}{k!}$$

where *P*(*k*) is the probability of *k* CMCs in a droplet and *λ* is the expected value of CMCs per droplet. In our study, the number of CMCs per sample typically ranged from 0 to 50, so Poisson statistics should apply. In the case of higher numbers of CMCs, Poisson would underestimate the total number, as some bubbles with a photoacoustic response would actually have two or more CMCs, but would be counted as one. However, even with 500 CMCs in a 1 ml sample resulting in 1000 bubbles, having two or more CMCs would have a probability of approximately 0.07.

Prior to each session of testing, we calibrated the photoacoustic system using PBS and 1 μm diameter black latex microspheres suspended in PBS. We ensured that running the photoacoustic system with PBS resulted in no photoacoustic waveforms, as there is no optical absorption in that case. When PBS samples resulted in errant photoacoustic responses, we replaced the detection chamber with a new one, as we assumed the previous one became contaminated. We then ran the system with 1 μm, black microspheres (Polysciences, Warrington, PA) to ensure that the photoacoustic responses were recorded, commensurate with the concentration of spheres. This process ensured that our system was functioning properly, minimizing false positive and negative signals.

Using this system, we tested 15 healthy volunteers. We separated their whole blood by a centrifugation process that included introduction of Histopaque 1077 to separate the buffy coat from the red blood cell layer. We also used red blood cell lysis and additional centrifugation to eliminate red blood cells, which would act as optical absorbers at 532 nm and thus provide false positive photoacoustic signals. All samples from healthy volunteers resulted in no photoacoustic detections.

### Patient Samples and Preparation

We assayed, in a blinded fashion, archived frozen blood buffy coat samples from 38 non-Stage IV melanoma patients who had been followed at the University of Pittsburgh Medical Center after definitive surgical management. Each sample comprised the buffy coat processed from 1 ml of whole blood. There were two to six samples for each patient. These were drawn at varying follow-up time points, over periods of up to 8 years from initial surgical excision of the primary melanoma lesions.

The 38 patients included 3 Stage I, 4 Stage II, and 27 Stage III individuals with melanoma of cutaneous primary lesions. Also included were four patients who had mucosal surface primary melanoma lesions. Blinded longitudinal clinical information was correlated with results of the blinded CMC assays, to examine whether detection of CMCs in Stage I–III patients was associated with eventual progression and development of metastatic disease. Five of the Stage III patients we studied had received treatment with adjuvant high dose interferon-α postoperatively. This treatment appeared to have no impact on our analysis. The number of days between initial surgical excision of each patient’s melanoma and detection of metastasis, for the patients whose disease progressed; or alternatively, between initial surgical excision and the last available blood sample for the patients who remained disease free; was determined by confidential blinded review of medical records. This number ranged from 102 to 2974 days.

Each blood sample was assayed by a standardized protocol. The samples had Histopaque 1077 (Sigma-Aldrich, St. Louis, MO) added prior to centrifugation and buffy coat extraction. Lysing buffer (BD Biosciences, Franklin Lakes, NJ) was also added to ensure removal of erythrocytes. Additionally, DNAase was added to eliminate free DNA fragments that could potentially bind cells together and interfere with acoustic flow cytometric enumeration of CMCs.

### CMC Numbers and Date of Metastasis

We compared the data of patient samples with greater than two CMCs that led to advanced disease to those from patients who remained disease-free. We plotted the number of CMCs per sample against the number of days after diagnosis. We did not use the data for patients with fewer than two CMCs as we wanted to determine if the population of patients with greater than two CMCs in any sample was distinct, depending on whether the patients advanced their disease or not. We determined the centroid of the two sets, then determined the Mahalanobis distance of each centroid to the total data. The Mahalanobis distance determines the distance of points to a reference in multiple dimensions, taking into account the covariance of the data [13]. As we computed the distance using the total data set, the covariance was computed over the entire set. This distance calculation was done to get a numerical indication whether the two data sets constituted different sets in the sense of cluster analysis.

## RESULTS

Data for each of the 38 patients in the study is shown in Tables 1, 2, and 3. These tables are arranged as patients with fewer than two CMCs in any sample, patients with greater than two CMCs in any sample who advanced to metastatic disease, and patients with greater than two CMCs in any sample who did not advance to metastatic disease, respectively. The initial stage of each patient is shown, along with the lowest and highest numbers of CMCs detected for all samples taken from each individual patient, over each patient’s indicated time of follow-up. The tables also indicate whether each patient progressed to metastatic disease; and for patients who did progress, the number of days from initial excision to the date metastatic disease was diagnosed. Alternatively, in patients who did not progress to metastatic disease, the table displays the number of days from their initial excision to their last blood sample assayed.

In our data set, failure to detect greater than two CMCs at all time points distinguished a population of patients in which none progressed to metastatic disease. No patients of the eleven who had two or fewer CMCs detected in all of their various time point samples progressed to metastatic disease. In contrast, 18 of the 27 patients (67%), who had greater than two CMCs detected on one or more time points, eventually progressed to metastatic disease during their individual times of follow-up. This categorical difference is statistically significant given a threshold of 0.05 on post hoc analysis by Fisher’s Exact Test, 2 × 2 contingency table; two-tailed *P* = 0.0002 (Table 4).

The mean and median number of days of follow-up observation for the 11 patients who always had two or fewer CMCs and never progressed to metastatic disease were 1288 and 1186 from the date of initial excision. For the 18 patients with greater than 2 CMCs on at least one occasion who progressed to metastatic disease, the mean and median number of days of observation from initial excision to diagnosis of metastatic disease were 850 and 727. For the nine patients with greater than 2 CMCs on at least one occasion who did not progress to metastatic disease, the mean and median number of days of follow-up from initial excision to their last tested sample were 728 and 495. Therefore, the observation of the categorical difference in incidence of progression to metastatic disease between the groups of patients with greater than 2 CMCs detected at any time point and two or fewer CMCs detected at all time points is not explainable as a result of difference in duration of follow-up time between the patient groups. The group with two or fewer CMCs on all occasions was observed an average of 438 days longer than the group with greater than 2 CMCs on at least one occasion who progressed to metastatic disease. The group with greater than 2 CMCs who did not progress to metastatic disease were followed an average of 122 days less than the group that progressed, with a median follow-up observation of 228 days less. This suggests that some of these patients may have progressed to metastatic dis-ease had they been observed longer. There was also a strong general trend for increasing rates of progression to metastatic disease with increasing thresholds for detected CMCs on at least a single occasion (Table 5).

While examining the CMC data in a converse manner, it becomes apparent that exceeding a low threshold of 2 CMCs/ml is a useful benchmark for predicting increased risk of developing metastatic disease. Detection of greater than 2 CMCs on any single occasion, up to detection of 100 or fewer CMCs on any occasion, was associated with a risk of development of metastatic disease in the low to mid 60% range. With detection of greater than 100 CMCs on any single occasion, the risk of metastasis was 71%; and with detection of greater than 200 CMCs on any single occasion, the risk of metastasis was 80% in the time frames during which these patients were observed.

In our entire data set, there were 18 metastatic patients out of a total of 38 patients, for an overall metastasis rate of 47.4% in the study. That overall metastasis rate is similar to the generally quoted 5-year approximately 50% metastasis rate for Stage III melanoma patients. This supports that the patient population in our study was generally representative of typical Stage III melanoma patients. That there were a few Stage IB and Stage II patients in the study population would be expected to skew the overall observed metastasis rate a bit below the standard quoted 50% rate for Stage III patients, which was in fact observed. Also, many of the patients were followed less than the standard 5-year benchmark, so one would expect a slightly lower overall metastasis rate than the historical figure of approximately 50% over 5 years for Stage III patients. Overall, our data set is consistent with the known historical metastasis rate and timeline of Stage III melanoma patients, supporting the validity and general applicability of our findings.

### CMC Numbers and Date of Metastasis

**Fig. 2.**
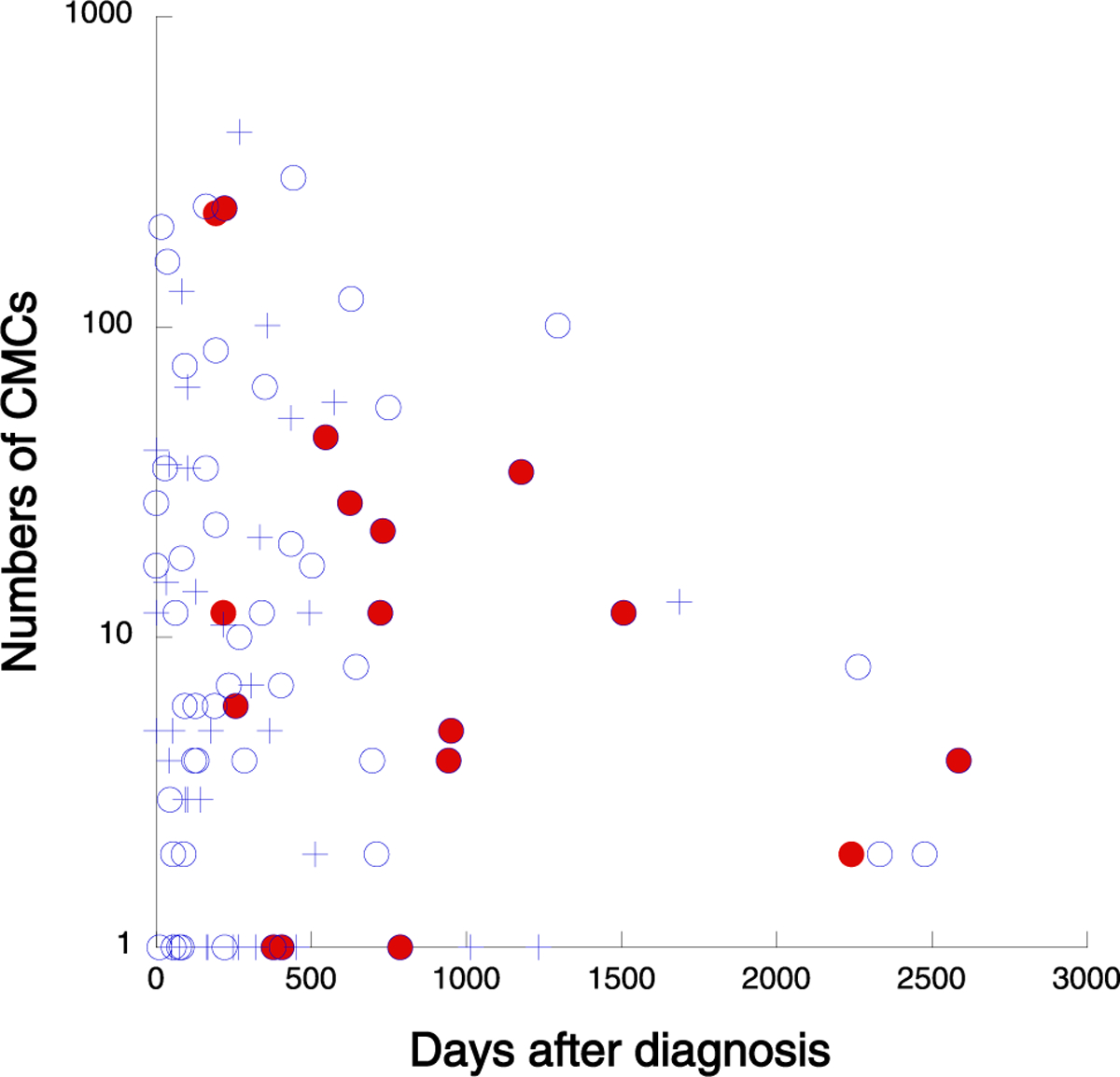
A plot of sample points from patients with at least one occasion of having two or more circulating melanoma cells (CMCs) detected. Plus signs are from those patients who were never diagnosed with metastasis. Samples from patients who did become metastatic are indicated by circles, with red circles specifically showing the day in which metastasis was diagnosed.

Figure 2 shows the data for patients who had greater than two CMCs on any given sample. For patients who did not advance to metastasis, the points are indicated by plus signs. For those patients who advanced to metastasis, the points are indicated by circles. The data point for which metastasis was found is indicated by a filled red circle. The Mahalanobis distance from the centroid of the nonmetastatic group to the whole set is 0.107, while the distance from the metastatic group is 0.216.

## DISCUSSION

Detection and analysis of markers in blood is a rich field of investigation for cancer research [16–19]. More specifically, detection and capture of CMCs in blood may aid in disease staging, monitoring and design of therapy, and prediction of metastasis of melanoma [20]. Our study, using photoacoustic flow cytometry to detect and enumerate CMCs, provides evidence of a low risk of progression to metastatic disease in patients whose CMCs never exceed a low threshold of 2 CMCs/ml. However, we would caution against using a single determination of CMCs from a patient’s blood sample to assess the risk for progression. It is possible for patients with initially low levels of CMCs to increase their number of CMCs to above the low threshold, below which we did not observe progression. We did observe progression to metastatic disease in a few patients who initially had no detectable CMCs. Therefore, continued repeated assay of blood for CMCs will be required for monitoring patients for early evidence of progression to metastatic disease. Our analysis only used CMC number as a predictor. It may be possible to develop a more highly predictive model that takes into account other factors, such as the trend of CMC numbers over time, and as well by genetic analysis of panels of individual CMCs, which can be captured and isolated by our photoacoustic system. However, the simple criterion of highest observed CMC number, as we used in this present analysis, has clinical utility and the advantage of simplicity. There might be an advantage in testing more blood volume, allowing better stratification of patient risk. This study tested buffy coats from 1 ml of blood. Taking a greater volume, such as 10 ml, is not burdensome to the patient and could yield additional useful CMC data, especially by capture and phenotypic or genetic analysis of panels of individual CMCs. While we did not analyze captured CMCs for this study, as the goal was to correlate CMC number with the onset of metastasis, photoacoustic flow cytometry is capable of capturing CMCs in suspension.

### Sensitivity of the Photoacoustic Flow Cytometer

This cell assay is not concentration dependent, unlike many chemical or biochemical assays. As long as there are pigmented cells of sufficient optical absorption in a volume of fluid, there will be a photoacoustic response when the cell passes through the laser beam. So one cell in a microliter will create a photoacoustic signal in our flow cytometer just as one cell in a liter would create such as signal. However, larger volume samples would result in more bubbles created by the two-phase flow process, increasing the amount of time needed for the assay. As the two-phase flow results in bubbles of approximately 1 μl using the flow and material parameters employed, this volume is the fundamental sampling unit that is relevant to our method. As the beam expands to cover the entire volume of each bubble, it detects a photoacoustic event in every bubble containing a CMC. Of course, pigmentation level of the cell is important, but this study showed that, given the natural pigmentation of CMCs, a predictive model of metastasis can be derived.

It must be noted that another photoacoustic technology, the Cytophone, developed by Vladimir et al. [21] and by Galanzha et al. [22], has achieved success in performing CMC counting *in vivo*. This accomplishment overcomes many challenges not encountered in our *in vitro* method, and potentially allows for testing of the entire blood volume of a patient. This advantage cannot be under-estimated, as much of the uncertainty of current CTC assays, including our own, comes from the limited volumes sampled.

### Benefit of Photoacoustic-Mediated Capture

**Fig. 3.**
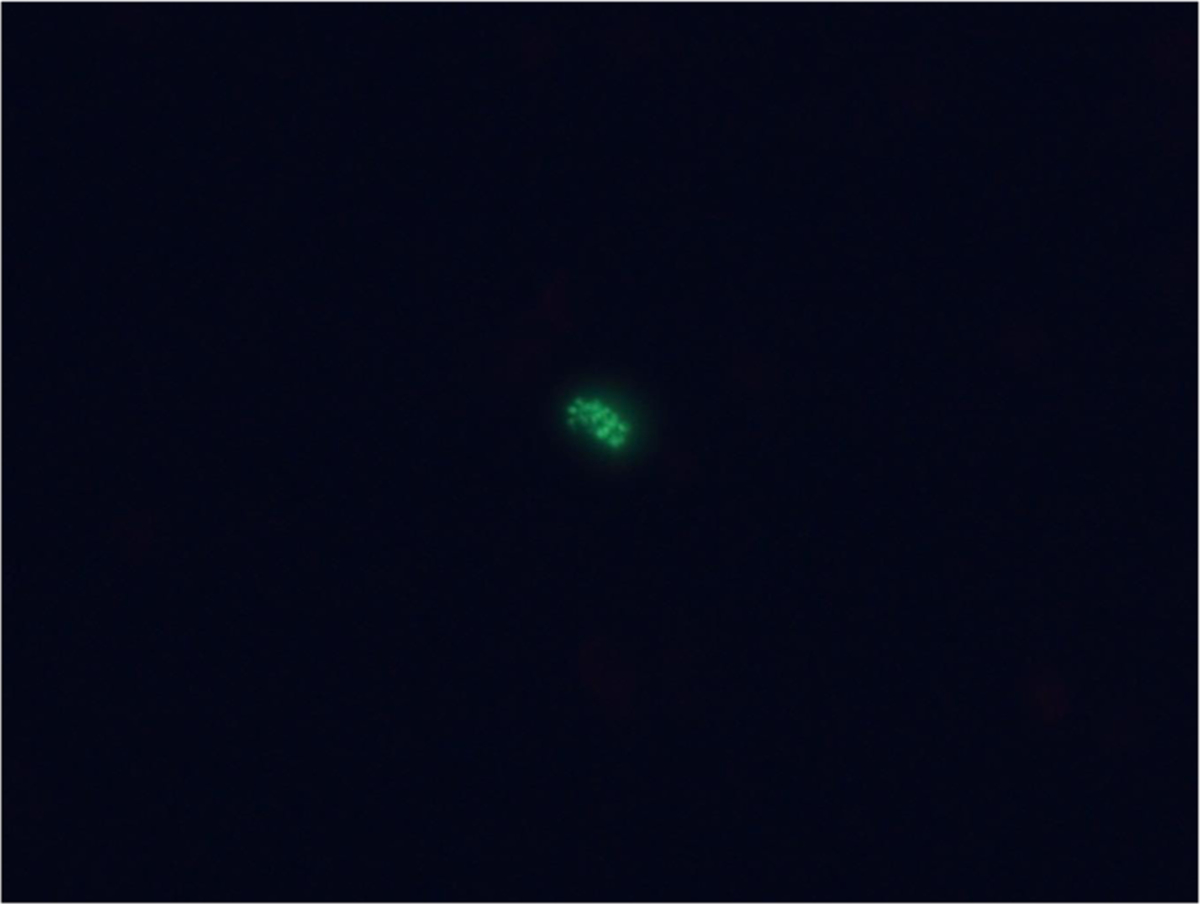
Green fluorescence indicates the presence of MART1 surface markers on a captured circulating melanoma cells from a Stage III melanoma patient.

We have used photoacoustic flow cytometry to perform immunocytochemical analysis, as shown in Figure 3, where green fluorescence illuminates MART1 antigens on the captured cell surface. We estimate that cell loss in the staining procedure could have been 20–50%. Further confounding fluorescent detection is the uncertainty of any given CMC expressing MART1. While imaging some fluorescent cells in the captured samples from photoacoustic flow cytometry gave some measure of validation that the method was effective in detecting CMCs, a formal study that includes such validation has numerous sources of error that would make it difficult to assess properly. However, this exploratory staining technique indicates that performing other molecular assays on captured cells is possible and could potentially be used for design of therapy, such as in testing for BRAF mutations [23,24].

### CMC Numbers and Date of Metastasis

Inspection of Figure 2 shows that most of the nonmetastatic cases, indicated by plus signs, occur early in the data set, mostly prior to about 500 days. While there are many points from the metastatic group prior to 500 days, there are many points later. More notable is that the majority of the samples where metastasis is detected occur after 500 days. This observation indicates that the nonmetastatic group might have developed metastasis if this group had been followed longer. Therefore, the threshold measurement of detecting more than two CMCs in any blood sample is a strong indicator of risk of metastasis.

Further indication that the two groups, metastatic and nonmetastatic, are actually in the same population is that the Mahalanobis distance is small. As this distance is not Euclidean, but accounts for the covariance in both dimensions, the values of 0.107 and 0.216 strongly indicate that both groups belong to the same, composite population.

### Nonpigmented Melanoma

Photoacoustic flow cytometry generates acoustic waves in melanoma cells due to the content of melanin pigment, which absorbs and transduces optical energy from laser pulses. However, some melanoma cells are nonpigmented or partially pigmented. Most melanomas are highly pigmented, with estimates of amelanotic melanoma being less than 5% [25] or 1.8–8.1%, though this latter figure includes partially pigmented melanoma [26]. We have developed means to enhance melanoma cell detection and induce nonpigmented cancer cells using antibody-labeled, exogenous optical absorbers, such as functionalized dyed microspheres [27,28]. Some researchers have found that undifferentiated and, hence, nonpigmented melanomas are responsible for disease with more metastatic potential. Muller et al. [29] stated that microphthalmia associated transcription factor (MITF), which is responsible for pigmentation, is relevant to prediction of metastatic potential. Specifically, MITF is known to block proliferation, so lack of MITF may result in nonpigmented CMCs that are more likely to lead to metastasis. However, while relying on pigmentation for photoacoustic signals, our results still provide valuable predictive value in a data set that was not selected beforehand for pigmentation. That is, in a population of patients, regardless of MITF expression, photoacoustic enumeration of CMCs still provides statistically significant classification of patients into metastatic and nonmetastatic groups.

### Future Work

Although we have restricted this model of disease progression to the simple, but straightforward, enumeration of the maximum number of CMCs in serial samples, we are planning a prospective study of melanoma patients to include other factors to incorporate a multivariate model for metastasis prediction. We will develop a survival model that uses CMC detection to provide estimates of model parameters that can be used to predict time to metastasis in early-stage melanoma patients. The time to metastasis will be modeled as

$$h\left(t\right)=h_{0}\left(t\right)e^{\left(\beta X_{ij}+\alpha Z_{i}\right)}$$

where *h*_0_(*t*) denotes a baseline function, *β* models the correlation of CMC number to time to metastasis, *α* denotes prior information about the patient, potentially including tumor burden or relevant blood chemistry levels. *Z*_*i*_ is the disease stage of the patient. *X*_*i*,*j*_ is a function derived from a longitudinal model that forms the basis for this model. Estimation of these parameters will yield the relationship of CMC numbers to advancement to metastastic disease and will provide a more sophisticated model for clinical prediction. Prior information about the patient may include sex, age, and treatment regimen. This study will be based on serial data from approximately 100 early-stage melanoma patients.

Furthermore, we have been investigating methods to improve classification of signals and hence, improving the predictive value of photoacoustic flow cytometry. In other work with photoacoustic signals, we used Bayesian methods to classify vascular and pigmented lesions in the skin [30]. We are developing unsupervised learning using convolutional and dense neural networks for feature extraction. This area of investigation offers rich developments in our ability to detect CTCs, but testing and validating the many possible cases is an ongoing process. In this study, however, we were able to extract notable inferences using the simple threshold classification explained in Materials and Methods.

## CONCLUSIONS

Photoacoustic flow cytometry was demonstrated to detect and quantify rare CMCs in blood samples of Stage I-III melanoma patients. In our data set, derived from serial blood draws from 38 Stage I–III melanoma patients, detection of 2 or fewer CMCs/ml of blood, on all occasions tested in an individual patient, was never associated with progression to metastatic disease. In contrast, detection of greater than 2 CMCs/ml, on even a single occasion, was statistically significantly associated with eventual progression to metastatic disease, though not all patients with greater than 2 CMCs had documented progression to metastatic disease over their varying duration individual follow-up periods. In addition, detection of CMCs at higher threshold levels, especially above 100 CMCs/ml, was associated with even higher rates of progression to metastatic disease. In summary, our results support that photoacoustic detection of CMCs in Stage I–III melanoma patients is useful for monitoring for early evidence of development of metastatic disease, which potentially may enable earlier and more effective therapeutic intervention.

## Figures and Tables

**TABLE 1.** Patients With Fewer Than Two Circulating Melanoma Cells (CMCs) on All Samples

Patient	Stage	Lowest CMC	Highest CMC	Metastasis	Days from diagnosis
1	3B	0	2	No	317
2	3B	0	2	No	152
3	Unknown	0	0	No	2889
4	3B	0	1	No	2974
5	1A	0	1	No	1186
6	3	0	1	No	2480
7	3A	0	1	No	358
8	1A	0	1	No	1876
9	2C	0	1	No	102
10	2B	0	2	No	1583
11	3B	0	2	No	251

**TABLE 2.** Patients With Greater than Two Circulating Melanoma Cells (CMCs) in Any Sample Who Advanced to Metastatic Disease

Patient	Stage	Lowest CMC	Highest CMC	Metastasis	Days from diagnosis
12	3	2	61	Yes	192
13	3B	0	16	Yes	212
14	3B	3	209	Yes	1852
15	3C	3	54	Yes	730
16	2C	0	19	Yes	951
17	3	0	7	Yes	786
18	Mucosal	0	34	Yes	266
19	1B	11	301	Yes	723
20	3B	43	234	Yes	547
21	2A	1	3	Yes	1670
22	3A	1	74	Yes	405
23	3A	7	83	Yes	624
24	3	1	3	Yes	2240
25	3C	34	240	Yes	220
26	Mucosal	33	100	Yes	934
27	Mucosal	3	161	Yes	1506
28	3B	0	5	Yes	378

**TABLE 3.** Patients With Greater Than Two Circulating Melanoma Cells (CMCs) in any Sample Without Ad-vancing to Metastatic Disease

Patient	Stage	Lowest CMC	Highest CMC	Metastasis	Days from diagnosis
29	3C	2	63	No	383
30	3B	11	129	No	213
31	3B	0	4	No	468
32	3C	4	100	No	2490
33	3C	0	56	No	1233
34	3A	0	50	No	435
35	3B	1	423	No	513
36	3A	0	11	No	495
37	3A	0	13	No	205
38	3B	2	4	No	468

**TABLE 4.** The 2 × 2 Contingency Table Showing Metastasis With Respect to the Circulating Melanoma Cell (CMC) Enumeration Threshold. The *P* Value Was Cal-culated as *P* = 0.0002

	≤2 CMCs	>2 CMCs
Metastasis	0	18
No metastasis	11	9

**TABLE 5.** The Progression of Circulating Melanoma Cell (CMC) Number Corresponding to Eventual Metastasis

Number of CMCs	Number that metastatic	Percentage metastatic
2 or fewer CMCs in all samples	0 of 11	0
3 or fewer CMCs in all samples	2 of 13	15
4 or fewer CMCs in all samples	3 of 15	20
5 or fewer CMCs in all samples	4 of 16	25
7 or fewer CMCs in all samples	5 of 17	29
19 or fewer CMCs in all samples	7 of 21	33
50 or fewer CMCs in all samples	8 of 22	36.4
100 or fewer CMCs in all samples	13 of 31	41.9
